# Analysis of the Involvement of the Isoleucine Biosynthesis Pathway in Photoheterotrophic Metabolism of *Rhodospirillum rubrum*

**DOI:** 10.3389/fmicb.2021.731976

**Published:** 2021-09-21

**Authors:** Guillaume Bayon-Vicente, Elie Marchand, Jeson Ducrotois, François E. Dufrasne, Regis Hallez, Ruddy Wattiez, Baptiste Leroy

**Affiliations:** ^1^Laboratory of Proteomics and Microbiology, Research Institute for Biosciences, University of Mons, Mons, Belgium; ^2^Bacterial Cell Cycle & Development (BCcD), Biology of Microorganisms Research Unit (URBM), Namur Research Institute for Life Science (NARILIS), University of Namur, Namur, Belgium; ^3^Namur Research College (NARC), University of Namur, Namur, Belgium; ^4^WELBIO, University of Namur, Namur, Belgium

**Keywords:** purple bacteria, acetic acid, photoheterotroph, redox balance, electron sink, isoleucine biosynthesis, light intensity, volatile fatty acids (VFA)

## Abstract

Purple non-sulfur bacteria (PNSB) are recognized as a highly versatile group of bacteria that assimilate a broad range of carbon sources. Growing heterotrophically, PNSB such as *Rhodospirillum rubrum* (*Rs. rubrum*) generate reduced equivalents that are used for biomass production. However, under photoheterotrophic conditions, more reduced electron carriers than required to produce biomass are generated. The excess of reduced equivalents still needs to be oxidized for the metabolism to optimally operate. These metabolic reactions are known as electron sinks. Most PNSB rely on the CO_2_-fixing Calvin cycle and H_2_ production to oxidize these reduced equivalents. In addition to these well-described electron sinks, the involvement of some pathways, such as polyhydroxyalkanoate (PHA) biosynthesis, in redox poise is still controversial and requires further studies. Among them, isoleucine biosynthesis has been recently highlighted as one of these potential pathways. Here, we explore the role of isoleucine biosynthesis in *Rs. rubrum*. Our results demonstrate that the isoleucine content is higher under illuminated conditions and that submitting *Rs. rubrum* to light stress further increases this phenomenon. Moreover, we explore the production of (p)ppGpp in *Rs. rubrum* and its potential link with light stress. We further demonstrate that a fully functional isoleucine biosynthesis pathway could be an important feature for the onset of *Rs. rubrum* growth under photoheterotrophic conditions even in the presence of an exogenous isoleucine source. Altogether, our data suggest that isoleucine biosynthesis could play a key role in redox homeostasis.

## Introduction

Purple non-sulfur bacteria (PNSB) constitute a metabolically highly versatile group of bacteria capable of assimilating a broad range of carbon sources. Among them, *Rhodospirillum rubrum* (*Rs. rubrum*) has been extensively studied for the assimilation of volatile fatty acids (VFAs). Among VFAs, acetate has received significant interest as this compound represents the most abundant VFA issued from fermentation processes (Ivanovsky et al., 1997; Erb et al., 2008, 2009; Berg and Ivanovsky, 2009; Leroy et al., 2015; Alloul et al., 2019; Bayon-Vicente et al., 2020a; De Meur et al., 2020). Although acetate assimilation has long been debated, it is now well established that acetate is mainly assimilated through the ethylmalonyl-CoA (EMC) pathway in isocitrate lyase-lacking organisms (*icl*^–^) (Erb et al., 2008; Leroy et al., 2015; De Meur et al., 2018). However, another metabolic cycle, the citramalate cycle, has, for a long time, been hypothesized as an alternative acetate assimilation pathway (Osumi and Katsuki, 1977; Ivanovsky et al., 1997; Berg and Ivanovsky, 2009). This cycle is characterized by the condensation of acetyl-CoA and pyruvate into citramalate [also called (*R*)-2-methylmalate] that is further converted into glyoxylate and propionyl-CoA (Berg and Ivanovsky, 2009). However, some enzymes required in the citramalate cycle operation have not been identified, suggesting that the observed early production of citramalate may have another function. Indeed, recent research carried out by our group showed that proteins involved in the isoleucine biosynthesis pathway are upregulated during the photoheterotrophic assimilation of acetate (Leroy et al., 2015; De Meur et al., 2018; Bayon-Vicente et al., 2020a), butyrate (De Meur et al., 2020), or valerate (Bayon-Vicente et al., 2020b) when compared to succinate. Moreover, our group has already shown that the abundance of free isoleucine was significantly higher in the presence of butyrate than in the presence of succinate (Ile/Arg_*but*_ = 10 *vs*. Ile/Arg_*Succ*_ = 2). This observation may explain the hypothesis of Ivanovsky’s group as citramalate or (*R*)-2-methylmalate constitutes the first intermediary of this pathway. As already suggested by other studies (Shimizu et al., 2010; Bayon-Vicente et al., 2020a; De Meur et al., 2020; McCully et al., 2020), isoleucine biosynthesis could act as an electron sink. In this context, isoleucine biosynthesis could be of major importance in redox homeostasis in order to deal with the redox imbalance triggered by non-favorable redox environmental conditions, such as the use of reduced carbon sources (Bayon-Vicente et al., 2020a, b; De Meur et al., 2020) or high light intensity (Bayon-Vicente et al., 2020a). Indeed, considering acetate as the sole source of carbon, the synthesis of isoleucine permits the net consumption of three reducing equivalents. Another argument corroborating this hypothesis is that the sudden increase in light intensity, another culture condition hypothesized to trigger redox imbalance, led to a comparable upregulation of enzymes of branched-chain amino acid (BCAA) biosynthesis in the presence of acetate, further suggesting the importance of BCAA synthesis in redox homeostasis (Bayon-Vicente et al., 2020a). Altogether, these data suggest that the isoleucine biosynthesis pathway could play a key role in redox homeostasis.

Alternatively, it was recently shown that a higher abundance of branched-chain amino acids could be the result of cellular stress triggering a stringent response. This stringent response is characterized by an increased production of intracellular signal molecules such as guanosine 5′-diphosphate, 3′-diphosphate (ppGpp), and guanosine 5′-triphosphate, 3′-diphosphate (pppGpp), collectively called (p)ppGpp or alarmones. This stringent response represents a strategy developed by bacteria to handle changing environmental conditions (Magnusson et al., 2003; Ronneau et al., 2016; Fang and Bauer, 2018). The spectrum of activity of (p)ppGpp has first been studied in chemotrophic organisms such as *Escherichia coli* (Magnusson et al., 2005; Eydallin et al., 2007), *Pseudomonas aeruginosa* (Erickson et al., 2004; Ruiz et al., 2004), or *Salmonella* (Pizarro-Cerdá and Tedin, 2004) and revealed that an accumulation of (p)ppGpp is involved in the biosynthesis of amino acids, in cell cycle control (Xiao et al., 1991; Beaufay et al., 2021), virulence gene expression (Erickson et al., 2004; Pizarro-Cerdá and Tedin, 2004), or biofilm formation (He et al., 2012). Interestingly, it was shown that *Rhodobacter capsulatus* adjusts the level of (p)ppGpp by controlling the Rel hydrolase activity in response to the intracellular branched-chain amino acid concentration (Fang and Bauer, 2018).

Here, we attempted to elucidate the role of the isoleucine biosynthesis pathway in *Rs. rubrum.* Firstly, we monitored the content of free isoleucine in *Rs. rubrum* under different culture conditions. Then, we decided to explore the regulation of isoleucine biosynthesis by stringent response by inspecting the production of (p)ppGpp in *Rs. rubrum* under different metabolic profiles. Finally, as isoleucine itself is described as an inhibitor of the biosynthesis pathway, we tested the addition of this amino acid to the culture medium to observe the effect of the inactivation of this potential electron sink on the ability of *Rs. rubrum* to grow with acetate as the sole carbon source.

## Materials and Methods

### Bacterial Strain, Medium Composition, and Cultivation Conditions

The wild-type and acetate competent strains of *Rs. rubrum* S1H (ATCC 25903) were cultivated in a defined medium as described previously (Leroy et al., 2015) under dark aerobic, light aerobic, and light anaerobic conditions. The acetate competent strain constitutes an acetate-acclimated strain characterized by a significant reduction of the lag phase (De Meur et al., 2018). Moreover, this strain has already shown outstanding tolerance to high light intensity (Bayon-Vicente et al., 2020a). Cultures were performed in 50-ml serum bottles filled with 40 ml medium. Concerning the dark and light aerobic conditions, the cultures were inoculated with a starting OD_680__nm_ = 0.1 and incubated at a temperature of 30°C under orbital shaking at 150 rpm. Photoheterotrophic light anaerobic cultures were inoculated at a starting OD_680__nm_ = 0.5 and incubated at 30°C at 180 rpm. The carbon concentration was set to 125 mM in terms of carbon (i.e., 62.50 mM for acetic acid and 31.25 mM for succinic acid). The medium was supplemented with 35 mM of ammonium chloride as the nitrogen source and 0.06 mM of biotin (final concentration). Moreover, depending on the experiment considered, the medium was supplemented with filtered 3 mM or 50 mM sodium bicarbonate (final concentration). The upper gaseous phase was flushed using pure N_2_ and the 50-ml flasks hermetically sealed. Cultures were subjected to 50 μmol photons/m^2^ s (10 W, 100 lm, 2,650 K; Sencys, Amsterdam, The Netherlands). To perform light stress experiments (see below), this intensity was elevated from 50 to 150 μmol photons/m^2^ s. Pre-cultures used for the different experiments were grown in the presence of succinate and acetate for the wild-type and acetate competent strains, respectively. Growth was monitored by measuring the optical density at 680 nm using a 1-cm path length cuvette and a Thermo Scientific Helios Zeta spectrophotometer (Waltham, MA, United States). When the optical density (OD) was higher than 1.0, the samples were diluted and the measured OD values were corrected for the dilution.

### Monitoring of the Acetate Consumption

Monitoring of the carbon source concentration was performed as described (Leroy et al., 2015). Culture supernatants were obtained through centrifugation at 12,000 rpm and stored at −20°C before analysis. One hundred microliters of the culture supernatants was analyzed by high-performance liquid chromatography (HPLC) refractometry (Waters 2695 Separation Module, WATERS^TM^, Milford, CT, United States; Waters 2414 Refractive Index Detector). The separation was realized in isocratic mode using a Shodex SUGAR SH1011 column (SHODEX^TM^, New York, NY, United States) (300 mm × 8 mm) with 5 mM H_2_SO_4_ as the mobile phase (flow rate = 1 ml/min). Detection was performed through refractometry. The carbon source concentration was assayed by integrating the carbon source-specific peak (RTacetate = 11.27 min) and based on a standard curve.

### Measurement of Amino Acid Abundance in the Biomass

Branched-chain amino acids were extracted from pellets issued from the centrifugation of 500 μl of culture. The pellet was resuspended in 1.5 ml methanol/chloroform solution (1:2, *v*/*v*). The resuspended pellet then underwent five freeze/thawing cycles, and 400 μl of Milli-Q water (Merck, Darmstadt, Germany) was then added and the mixture centrifuged (5,000 rpm, 10 min, 4°C). The upper aqueous phase was recovered and submitted to SpeedVac before being stored at −20°C until analysis. The obtained pellet was then resuspended in 0.2% (*v*/*v*) formic acid in ultrapure MS-grade water. The BCAA content was analyzed using an Eksigent LC425 system coupled to a Q-TRAP instrument (AB Sciex Q-Trap-6500+; ABSciex, Framingham, MA, United States) used in multiple reaction monitoring (MRM) mode. The amino acids were separated on a C18 YMC-Triat 0.3-mm × 150-mm column operated at a flow rate of 5 μl/min in isocratic mode [3% acetonitrile (*v*/*v*) and 1% formic acid (*v*/*v*)] for 5 min, followed by an acetonitrile gradient from 3 to 55% in 3 min. The following transitions were used to quantify the following amino acids: arginine 175/116 and isoleucine 132/69. To avoid extraction bias, isoleucine abundance was expressed as the ratio of the area under the curve for its specific transition to the area under the curve of the specific transition of the arginine.

### Detection of Intracellular (p)ppGpp Level

The (p)ppGpp levels were visualized as described previously (Ronneau et al., 2016), with some modifications. Briefly, bacteria were grown under dark aerobic, light aerobic, and light anaerobic conditions in P-free culture medium. Once cultures entered the exponential phase, 25 μl of KH_2_^32^PO_4_ was added at a final concentration of 100 μCi ml^–1^ and the cultures incubated for 1 h. Then 8 ml of the culture was centrifuged and used for (p)ppGpp extraction using 500 μl of 2 M formic acid, incubated on ice for 30 min, and then stored overnight at −20°C. The cell extracts were pelleted (14,000 rpm, 5 min) and 6 × 2 μl of the supernatant was spotted onto a polyethyleneimine (PEI) plate (Macherey-Nagel, Duren, Germany). The PEI plate was then developed in 1.5 M KH_2_PO_4_ (pH 3.4) at room temperature. Finally, the PEI plates were imaged on a MS Storage Phosphor Screen (GE Healthcare, Chicago, IL, United States) and analyzed with a Cyclone Phosphor Imager (PerkinElmer, Waltham, MA, United States). The ratio between ppGpp and GTP was analyzed using ImageJ software.

### 3-Methyl-2-oxopentanoate Extraction and Quantification

The methanolysis of 3-methyl-2-oxopentanoate was conducted as previously described (Bayon-Vicente et al., 2020a). Briefly, 500 μl of culture was centrifuged (8,000 rpm, 15 min) and stored at −20°C until analyzed. 3-Methyl-2-oxopentanoate was extracted and methanolyzed by resuspending the freeze-dried supernatant, respectively, in 500 μl of chloroform and 2 ml of methanolysis solution consisting of UHPLC methanol/concentrated HCl (90:10). The methanolysis solution also includes 0.1 mg/ml of 3-methylbenzoic acid as the internal standard. The mixture was then incubated at 100°C for 2 h and then cooled down on ice. One milliliter of distilled water was then added, and the bottom chloroform part was recovered and analyzed by GC-MS. The obtained spectrum was compared to the NIST05 ion spectrum bank. 3-Methyl-2-oxopentanoate content was expressed as arbitrary units (AU) corresponding to the area under the curve for the extracted ion chromatogram for *m*/*z* = 57 standardized to the dry cell weight.

### Acetolactate Synthase Activity Test

Cells were harvested at different growth phases (i.e., lag phase, early exponential phase, or late exponential phase) before being centrifuged and washed using phosphate buffer (50 mM, pH 7.0). Cells were lysed in 100 μl phosphate buffer using 25 mg of glass bead (bead size, < 106 μm; Sigma-Aldrich, St. Louis, MO, United States) and lysozyme (final concentration, 1 mg/ml). Cell-free extracts were obtained by centrifugation (13,000 rpm, 10 min, 4°C) and the protein concentration was determined using the Bradford method (Bradford, 1976), with bovine gamma globulin as a standard. Acetolactate synthase activity was examined as described previously (Muhitch, 1988). The acetolactate produced after 1 h was assayed at a single end point by conversion to acetoin, which was detected by the reaction of Westerfeldt (Westerfeldt, 1945) and quantified through the use of a standard curve after subtraction of the acetoin produced without substrate. Acetoin content was then normalized by the protein content.

## Results and Discussion

### Impact of Volatile Fatty Acids and Metabolic Regime on Relative Isoleucine Abundance

**FIGURE 1:**
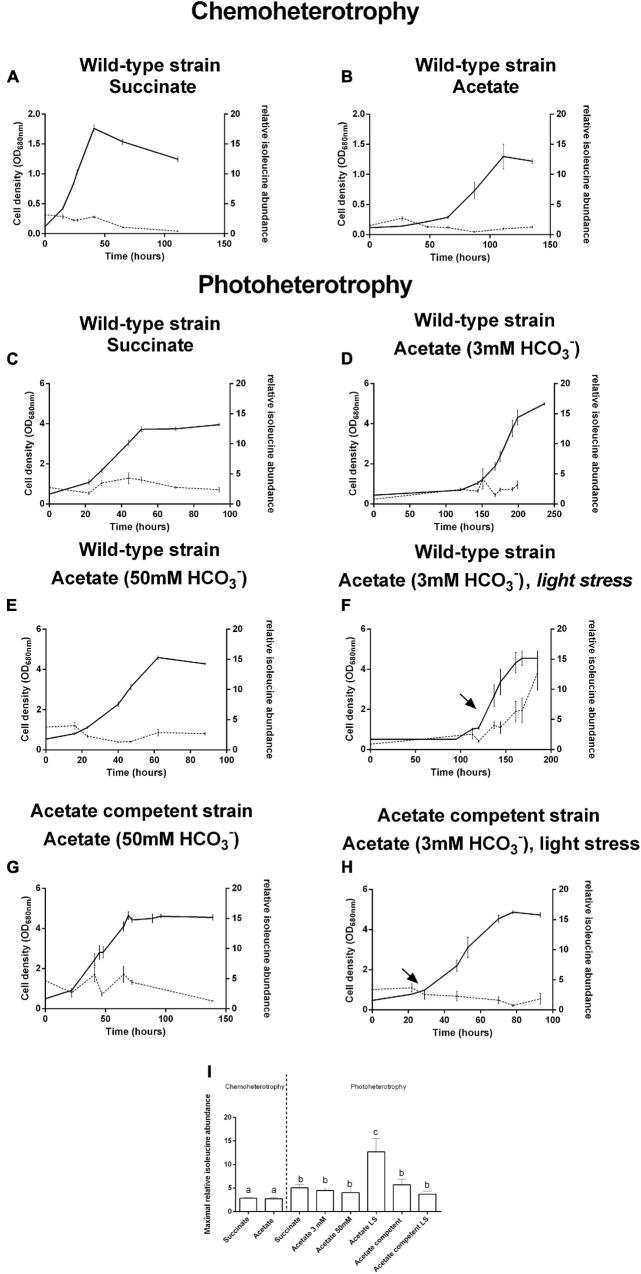
Growth (*full lines*) and relative isoleucine abundance (*dotted lines*) of *Rhodospirillum rubrum* wild-type **(A–F)** and acetate competent **(G,H)** strains in the presence of succinate **(A,C)** or acetate **(B,D–H)** under the chemoheterotrophic **(A,B)** or the photoheterotrophic **(C–H)** regime. *Arrows* represent light stress. Relative isoleucine abundance is expressed as the ratio between the area under the curve obtained in multiple reaction monitoring (MRM) of the isoleucine trace and the area under the curve of arginine. Maximal relative isoleucine abundances are aggregated in **(I)**. *N* = 5. Results are represented as the mean ± SEM. *Lowercase letters* in **(I)** represent statistical groups (*t*-test: *p* < 0.05).

Isoleucine biosynthesis-related enzymes have already been highlighted as upregulated in the presence of VFAs (Leroy et al., 2015; De Meur et al., 2018, 2020; Bayon-Vicente et al., 2020b). Moreover, the relative abundance of isoleucine has already been demonstrated to be higher in the presence of butyrate than in the presence of succinate (De Meur et al., 2020). Thus, we decided to monitor all along the growth curve the relative abundance of isoleucine in the presence of succinate or acetate as the sole carbon source, under photoheterotrophic and chemoheterotrophic conditions. As the fixation of CO_2_ has already been demonstrated to act on redox homeostasis (Gordon and McKinlay, 2014), we decided to also investigate the impact of the addition of 50 mM HCO_3_^–^ on isoleucine content. This relative abundance was calculated as the ratio of the abundance obtained for isoleucine to the one obtained for arginine, as previously described (De Meur et al., 2020). Arginine was chosen as its abundance remains stable over the growth curve (Supplementary Table 1). Moreover, we have already conducted several proteomic studies highlighting the impact of the different VFAs on the isoleucine biosynthesis pathway, but none showed that the arginine biosynthesis pathway was impacted by our conditions (i.e., the use of VFAs and/or light stress) (Leroy et al., 2015; Bayon-Vicente et al., 2020a, b; De Meur et al., 2020). No significant difference was observed regarding the profile of isoleucine abundance along the growth curve whatever the metabolic regime, the carbon source, or the strain tested. We decided to compare the highest abundance reached during the growth curve for all the conditions. Interestingly, we observed that the relative isoleucine abundance always reached a significantly higher level under the photoheterotrophic regime than under the chemoheterotrophic regime (2.5-fold higher at the end of the culture; *t*-test: *p* < 0.05) (Figure 1). On the other hand, no difference was observed between the relative isoleucine abundances for bacteria cultivated in the presence of acetate or succinate under both metabolic regimes. It is interesting to note that the supplementation of the medium with 50 mM NaHCO_3_ did not result in a modification of the isoleucine content. Moreover, differences were never observed between the acetate competent strain and the wild-type strain cultivated in the presence of acetate. This observation suggests that the higher redox stress tolerance observed for the acetate competent strain is not linked to a higher flux through isoleucine biosynthesis. As a sudden increase in the light intensity (light stress) has already been linked to the upregulation of enzymes of the isoleucine biosynthesis pathway (Bayon-Vicente et al., 2020a), we also studied the relative abundance of isoleucine after such a sudden increase in light intensity. Very interestingly, the application of light stress to bacteria cultivated in the presence of acetate led to a significant sixfold increase (*t*-test: *p* < 0.05) in the cellular isoleucine content (Figure 1). This observation corroborates the results obtained in our previous research that showed an upregulation of the enzymes involved in isoleucine biosynthesis following an increase in light intensity (Bayon-Vicente et al., 2020a). It is interesting to note that this result has not been observed for the acetate competent strain submitted to light stress, further highlighting that the outstanding tolerance of this strain to light stress does not rely on isoleucine synthesis.

**FIGURE 2:**
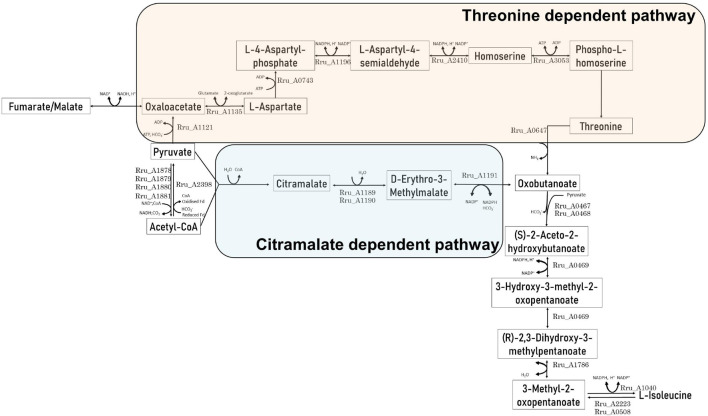
Schematic representation of the citramalate-dependent and threonine-dependent isoleucine biosynthesis pathways in the presence of acetate as the carbon source.

The present results suggest that the isoleucine biosynthesis pathway could be used in order to regenerate the reduced cofactors synthetized through the reverse activity of NADH dehydrogenase (Klamt et al., 2008; Golomysova et al., 2010) after light stress in the presence of acetate. In *Rs. rubrum*, isoleucine biosynthesis is sustained through two pathways: whereas the first one relies on the threonine biosynthesis pathway (McCully et al., 2020), the second one is linked to citramalate synthesis (Leroy et al., 2015). The former has already been linked to redox homeostasis by McCully and collaborators (McCully et al., 2020). However, although some clues seem to indicate that the latter could be linked to redox homeostasis, no clear evidence has been brought forward. Nevertheless, although McCully and collaborators have suggested that the biosynthesis of isoleucine through the citramalate pathway constitutes a NAD^+^ reducing pathway in the Calvin–Benson–Bassham cycle mutant, this statement was related to experiments done with fumarate as the carbon source (McCully et al., 2020). However, considering acetate as the carbon source, the biosynthesis of isoleucine through citramalate allows the net consumption of three reduced equivalents (Figure 2). It is interesting to note that, depending on the substrate, different pathways leading to isoleucine biosynthesis are used. Indeed, in the case of the study of McCully et al., in the presence of fumarate, isoleucine is suggested to be synthetized through a threonine-dependent pathway (Figure 2) (McCully et al., 2020), whereas our group, in the presence of acetate as the carbon source, highlighted a citramalate-dependent pathway (Figure 2) (Leroy et al., 2015; Bayon-Vicente et al., 2020a). Moreover, although the use of the threonine-dependent isoleucine pathway in the presence of acetate would represent a reduced equivalent consuming pathway, this pathway would also constitute a HCO_3_^–^ consuming pathway. Indeed, the production of oxobutanoate in the presence of acetate is accompanied by the net consumption of two molecules of HCO_3_^–^ (Figure 2). Considering the low concentrations of the bicarbonate ions in our culture medium (i.e., 3 mM), this pathway would constitute an unfavorable one when compared to the citramalate-dependent pathway (i.e., no net consumption of HCO_3_^–^). It is thus interesting to note that both mentioned pathways could constitute redox balancing pathways and that, depending on the nutritional context, *Rs. rubrum* would be able to switch from one to another.

### Impact of Volatile Fatty Acids and Metabolic Regime on ppGpp Accumulation

Although a high intracellular branched-chain amino acid concentration was already linked to a reduced (p)ppGpp content (Fang and Bauer, 2018), another study conducted on *E. coli* has already stated that (p)ppGpp accumulation could lead to an upregulation of the amino acid biosynthesis genes (Paul et al., 2005). Moreover, it was already demonstrated in *R. capsulatus* that the product of a single gene, *rel*, regulates the accumulation of (p)ppGpp (Mittenhuber, 2001). In 2004, Masuda and Bauer demonstrated a link between Rel activity and HvrA, a *trans*-acting regulatory protein, and demonstrated that *rel* can only be deleted if *hvrA* was knocked out first. HvrA has also been recognized as implicated in the activation of *puf* operon, which encodes for the α- and β-polypeptides of the B875nm complex (Masuda and Bauer, 2004), which could explain the observed higher content under photoheterotrophic conditions. Thus, it could be hypothesized that an overexcitation of the photopigment, as is the case under light stress, would result in the activation of HrvA, further leading to the accumulation of (p)ppGpp, which in turn results in amino acid overproduction. Altogether, the above-mentioned studies depict a precise regulation loop. Thus, the increase in isoleucine content could be linked to either a stringent response just after light stress or due to the implication of the isoleucine biosynthesis pathway in redox homeostasis.

In order to first explore whether the higher isoleucine content observed previously could be linked to the onset of a stringent response, we investigated the impact of the different metabolic regimes and of the sudden light increase on (p)ppGpp detection. As stringent response constitutes a quick answer to an environmental stimulus, its investigation must be performed in a reduced time frame after the stimulus. Hence, samples for (p)ppGpp quantification were taken at the beginning of the exponential phase for the chemoheterotrophic and photoheterotrophic conditions or 1 h after the light stress. To distinguish the potential effect of the anaerobic condition for illumination, we also performed (p)ppGpp quantification in the light aerobic condition. To evaluate (p)ppGpp accumulation in the different conditions, we spotted a positive and a negative control corresponding to extracts of a wild-type strain and a Δ*rel* strain of *Caulobacter crescentus*, respectively.

**FIGURE 3:**
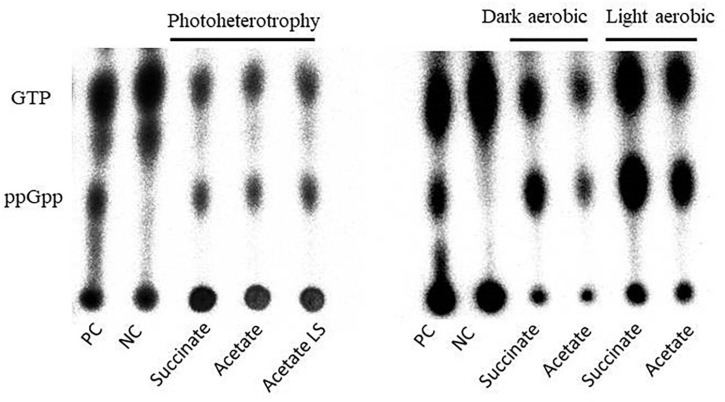
Autoradiography of polyethyleneimine (PEI) thin-layer chromatography of formic acid cell extract from *Caulobacter crescentus* (*PC* and *NC*) and *Rhodospirillum rubrum* (succinate and acetate) cultivated under photoheterotrophic (*left panel*) or dark or light aerobic (*right panel*) conditions.

Interestingly, each tested condition led to ppGpp accumulation, suggesting that the metabolic regimes seemed to have no effect on this accumulation (Figure 3). To further investigate the ppGpp accumulation in *Rs. rubrum*, we computed the ratio between ppGpp and GTP. However, no significant difference has been observed between the tested conditions (Table 1), further confirming that the differential relative isoleucine abundance after light stress cannot be, at least entirely, explained by an accumulation of ppGpp and, thus, to a stringent response of *Rs. rubrum*. Moreover, no difference in the ppGpp accumulation was observed between bacteria cultivated under the chemoheterotrophic and the photoheterotrophic condition.

**TABLE 1:** Signal left by ppGpp and GTP molecules following autoradiography of polyethyleneimine (PEI) thin-layer chromatography of formic acid cell extract from *Rhodospirillum rubrum* computed by ImageJ software.

**Conditions**	**ppGpp signal**	**GTP signal**	**ppGpp/GTP**
**Chemoheterotrophy**			
Succinate dark aerobic	7,454.639	9,141.287	0.815
Acetate dark aerobic	4,191.585	5,186.420	0.808
Succinate light aerobic	13,819.397	17,162.500	0.805
Acetate light aerobic	9,744.183	12,174.929	0.800
**Photoheterotrophy**			
Succinate light anaerobic	7,979.603	9,932.914	0.803
Acetate light anaerobic	7,654.437	9,422.640	0.812
Acetate light stress	7,476.207	9,245.424	0.809

*The third column represents the normalized data described as the ratio between the ppGpp and GTP signals.*

### Impact of the Addition of Isoleucine on Photoheterotrophic Assimilation of Acetate

**FIGURE 4:**
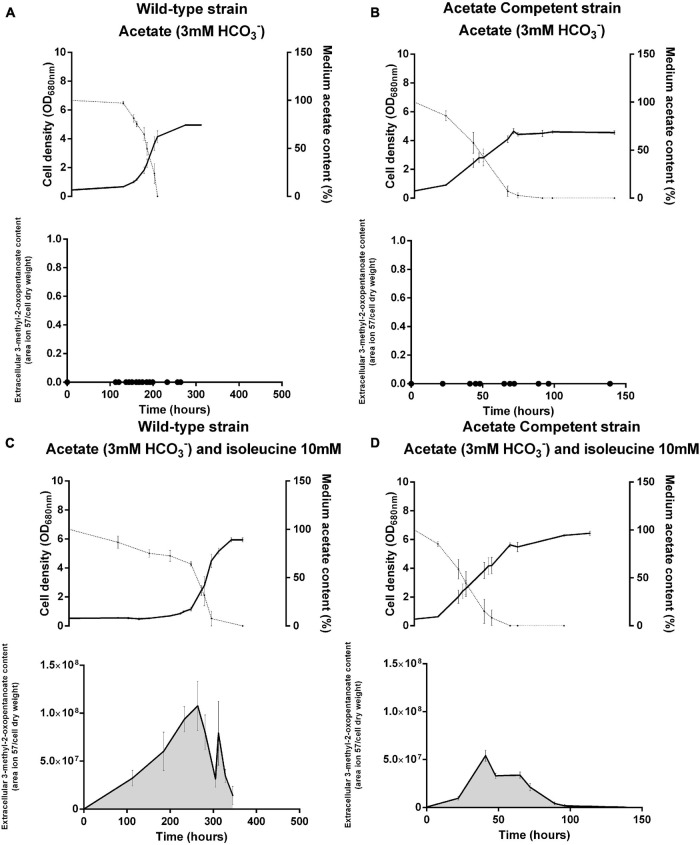
*Rhodospirillum rubrum* wild-type **(A,C)** and acetate competent **(B,D)** strains cultivated in the presence of acetate with **(C,D)** or without **(A,B)** 10 mM of isoleucine. *Upper panels* represent the growth (*full lines*) and acetate consumption (*dotted lines*) and *lower panels* represent the content of 3-methyl-2-oxopentanoate in the medium. *N* = 5. Results are represented as the mean ± SEM.

The higher content of isoleucine seems to be an important feature of the phototrophic metabolism that is further exacerbated after light stress in the presence of acetate. The biosynthesis of BCAAs was already hypothesized to act as an electron sink (Shimizu et al., 2010; McCully et al., 2020). Thus, in order to investigate the potential impact of isoleucine biosynthesis on redox homeostasis, we attempted to inhibit this biosynthetic pathway by adding 10 mM of isoleucine (Tanaka, 2003; Elišáková et al., 2005) in the medium of bacteria cultivated in the presence of acetate. As the acetate competent strain of *Rs. rubrum* has already been identified as particularly tolerant to redox stress (De Meur et al., 2018; Bayon-Vicente et al., 2020a), we also studied the phenotypic response of this strain to the addition of isoleucine. Interestingly, the growth of the wild-type strain in the presence of 10 mM isoleucine was characterized by a remarkable lag phase lasting for more than 250 h, which was not observed when the wild-type strain was cultivated in the absence of isoleucine (Figure 4). It is interesting to note that the phenotype of the acetate competent strain was not impacted by the addition of isoleucine and that no lag phase was observed for this strain (Figure 4). The lag phase observed during the photoheterotrophic assimilation of acetate has already been associated with redox stress linked to a high light/cell ratio (Leroy et al., 2015). This redox stress could be reduced by the addition of HCO_3_^–^ in the medium or an increase in the inoculum size. This suggests that the isoleucine biosynthesis pathway may act as an electron sink helping cells balance the redox stress and accelerating the onset of growth under the photoheterotrophic regime in the presence of acetate. However, in both strains, a comparable OD_680__nm_ of about 7 in the presence of isoleucine is reached, involving similar biomass being produced from the available carbon source, which is not the case in the absence of isoleucine, where an OD_680__nm_ of about 5 is reached. Curiously, whereas the exhaustion of acetate is associated with the end of the exponential phase in the presence of acetate as the sole source of carbon, the growth of *Rs. rubrum* in the presence of isoleucine continued after the total consumption of acetate. This observation suggests that bacterial growth is sustained by isoleucine or one of the degradation products utilized after acetate consumption, but as isoleucine quantitation has not been performed in the supernatant, this hypothesis cannot be confirmed (Figure 4).

Interestingly, the GC-MS analysis of the cell-free medium revealed the emergence of the molecule 3-methyl-2-oxopentanoate in cultures grown in the presence of isoleucine. This compound is known to result from the deamination of isoleucine and constitutes the first intermediary of the isoleucine degradation pathway (Figure 2). This compound is absent from cultures grown in the absence of isoleucine (Figure 4) and in the inoculum-free flask (data not shown). It demonstrates that this compound is linked to the presence of isoleucine in the medium and probably reflects isoleucine degradation by *Rs. rubrum*. Interestingly, for the wild-type strain, the peak of 3-methyl-2-oxopentanoate is observed just before the onset of growth, which may suggest that isoleucine degradation into 3-methyl-2-oxopentanoate is a prerequisite of photoheterotrophic growth in the presence of acetate.

Considering the potential inhibitory effect of isoleucine on the BCAA synthesis pathway, our observations indicate that the isoleucine biosynthetic pathway could be essential to balancing the redox stress associated with the lag phase. The degradation of isoleucine would then release this inhibition, allowing the onset of growth. Interestingly, no lag phase was observed for the acetate competent strain. Moreover, 3-methyl-2-oxopentanoate appeared to be more abundant in the wild-type strain than in the acetate competent strain.

**FIGURE 5:**
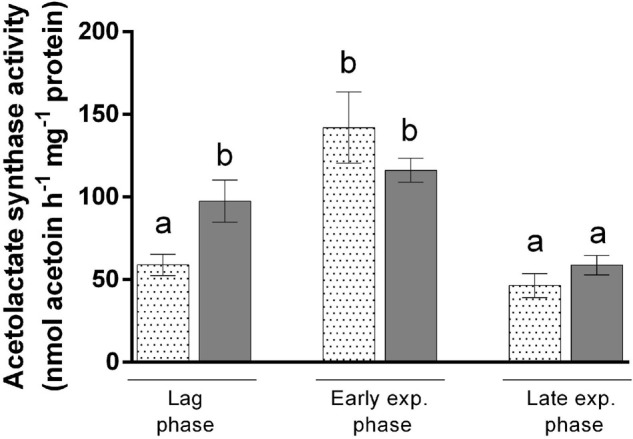
Enzymatic activity assay of acetolactate synthase issued from the cell extract of *Rhodospirillum rubrum* cultivated in the presence of acetate and 3 mM HCO_3_^–^ with (*dotted bars*) or without (*filled bars*) 10 mM isoleucine. *N* = 5. Results are represented as the mean ± SEM. *Lowercase letters* represent statistical groups (*t*-test: *p* < 0.05).

To attest the reduced flux through the isoleucine biosynthetic pathway when this amino acid is present in the medium, we measured the activity of acetolactate synthase in bacteria grown with acetate in the presence or absence of isoleucine. Acetolactate synthase is involved in the first step of BCAA synthesis (Rru_A0467 and Rru_A0468) (Figure 2) and was already highlighted by proteomic analyses in several studies (Leroy et al., 2015; Bayon-Vicente et al., 2020a). This enzyme is shared between the leucine, valine, and isoleucine biosynthesis pathway and is known to catalyze the conversion of two molecules of pyruvate into one molecule of (*S*)-acetolactate, which is one of the precursors of valine and leucine. However, in the presence of 2-oxobutanoate and pyruvate, a molecule of (*S*)-2-aceto-2-hydroxybutanoate is formed. This molecule is the precursor of isoleucine (Leroy et al., 2015). To follow the activity along the growth, we performed an enzymatic assay during the different growth phases and compared it to cultures that were not submitted to isoleucine inhibition in the same steps of the growth phase (end lag phase: OD_680__nm_ = 0.5; early exponential phase: OD_680__nm_ = 1.5; and late exponential phase: OD_680__nm_ = 4.25). Interestingly, an activity of acetolactate synthase has been detected in bacteria cultivated in the presence of isoleucine during the lag phase, although a 1.73-fold reduction in the activity was observed in comparison to cultures performed without isoleucine (*t*-test: *p* < 0.05) (Figure 5). Moreover, this activity increased during the early exponential phase, reaching activity comparable to the one observed without isoleucine. Therefore, we have shown that isoleucine displays an inhibitory effect on the acetolactate synthase activity that has been released before the exponential phase. These observations corroborate the 3-methyl-2oxopentanoate quantitation and further suggest that a fully functional isoleucine biosynthesis pathway is necessary for the onset of growth during the photoheterotrophic assimilation of *Rs. rubrum* in the presence of acetate. Interestingly, measurement of the activity of acetolactate synthase without added isoleucine revealed that the activity of this enzyme is significantly higher during the lag and early exponential phases than that during the late exponential phase (*t*-test: *p* < 0.05). The early phases of growth are characterized by a high light/cell ratio that has already been shown to trigger redox stress through the reverse activity of NADH dehydrogenase (Klamt et al., 2008; Golomysova et al., 2010; Bayon-Vicente et al., 2020a). During the late exponential phase, the light/cell ratio decreases and redox stress is reduced. Therefore, it appears that the isoleucine biosynthesis pathway plays a role in redox homeostasis by consuming the excess of reduced power, as already hypothesized (Shimizu et al., 2010; Bayon-Vicente et al., 2020a; De Meur et al., 2020; McCully et al., 2020). In that context, the synthesis of isoleucine may act as an electron sink. Altogether, these results strongly suggest an implication of the isoleucine biosynthesis pathway in redox balance homeostasis.

**FIGURE 6:**
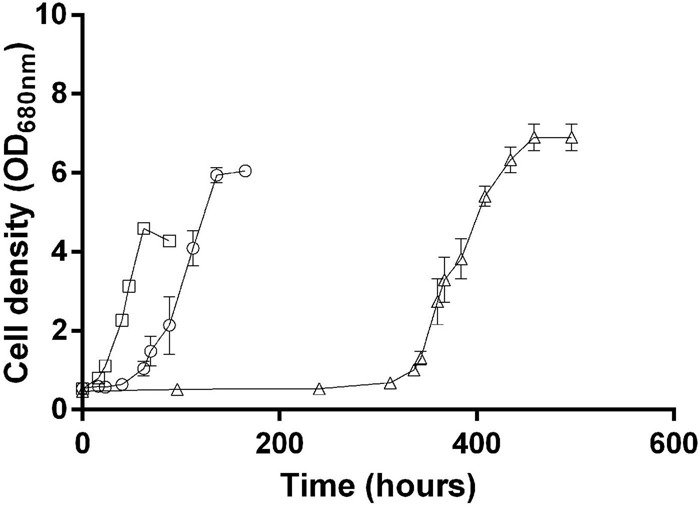
*Rhodospirillum rubrum* growth in the presence of acetate with 3 mM HCO_3_^–^ supplemented with 10 mM isoleucine (*open triangle*) or with 50 mM of HCO_3_^–^ supplemented (*open circle*) or not (*open square*) with 10 mM isoleucine. *N* = 5. Results are represented as the mean ± SEM.

Based on these observations, we hypothesize that the synthesis of isoleucine, which is inhibited by the presence of isoleucine in the medium, helps cells balance the redox stress responsible for the lag phase and the late onset of growth when *Rs. rubrum* is inoculated in acetate-containing medium. To verify this hypothesis, we cultivated *Rs. rubrum* in the presence of acetate and isoleucine, but in medium supplemented with 50 mM HCO_3_^–^, as it has been previously shown to shorten the duration of the initial lag phase (Bayon-Vicente et al., 2020a) and that the fixation of bicarbonate ions is well documented to act as an electron sink (McKinlay and Harwood, 2010; Wang et al., 2010; Rizk et al., 2011; Gordon and McKinlay, 2014). The addition of 50 mM HCO_3_^–^ led to a clear shortening of the lag phase in the presence of isoleucine (lag phase isoleucine + 3 mM HCO_3_^–^, ∼300 h; lag phase isoleucine + 50 mM HCO_3_^–^, ∼50 h) (Figure 6). Considering the electron sink role of CO_2_ fixation (McKinlay and Harwood, 2010, 2011; Rizk et al., 2011; Gordon and McKinlay, 2014), this result suggests that the isoleucine biosynthesis pathway could be considered as an electron sink in *Rs. rubrum.* Moreover, it is interesting to note that, although the lag phase is shorter in the presence of 50 mM HCO_3_^–^ than in the presence of 3 mM HCO_3_^–^, an unusual lag phase was still observed. It suggests that, although the addition of bicarbonate ions helped mitigate the excess of the reducing equivalent, inhibition of the isoleucine biosynthesis pathway by isoleucine still had a substantial impact on the growth of *Rs. rubrum*.

## Conclusion

The data reported here are supported by previous studies conducted by several groups (Shimizu et al., 2010; Leroy et al., 2015; Bayon-Vicente et al., 2020a, b; De Meur et al., 2020; McCully et al., 2020) and indicate that *Rs. rubrum* could use the isoleucine biosynthesis pathway to help maintain redox homeostasis during photoheterotrophic metabolism. Indeed, we showed that a sudden increase of light intensity from 50 to 150 μmol photons/m^2^ s was responsible for the increase in isoleucine abundance in the wild-type strain, but not in the acetate competent strain, which is known to be highly tolerant to light stress. Moreover, we also showed a slight but significant difference in the isoleucine content between bacteria grown under photoheterotrophic and chemotrophic conditions (*t*-test: *p* < 0.05). These observations suggest that the isoleucine biosynthesis pathway could be of major importance for growth under photoheterotrophic conditions. However, our results also demonstrate that this increase in isoleucine content was not linked to a general stress triggering a stringent response. Finally, based on the quantitation of 3-methyl-2-oxopentanoate and enzymatic assays in the presence or absence of isoleucine, we showed that a functional isoleucine biosynthesis pathway constitutes a key element for the onset of growth during the photoheterotrophic assimilation of acetate. Altogether, our results suggest that isoleucine biosynthesis could play a major role in redox homeostasis and could thus be considered as an alternative electron sink for purple bacteria when growing photoheterotrophically.

## Data Availability Statement

The raw data supporting the conclusions of this article will be made available by the authors, without undue reservation.

## Author Contributions

GB-V, RW, and BL designed the study. GB-V and JD performed the *Rs. rubrum* cultivation experiments. GB-V and BL designed the mass spectrometry analysis. GB-V and JD performed the mass spectrometry analyses and the bioinformatics analysis. GB-V, EM, and RH designed the (p)ppGpp quantification experiment. GB-V and EM conducted the (p)ppGpp quantification experiment. GB-V, JD, and FD wrote the manuscript. FD, EM, RH, RW, and BL revised the manuscript. All authors contributed to the article and approved the submitted version.

## Conflict of Interest

The authors declare that the research was conducted in the absence of any commercial or financial relationships that could be construed as a potential conflict of interest.

## Publisher’s Note

All claims expressed in this article are solely those of the authors and do not necessarily represent those of their affiliated organizations, or those of the publisher, the editors and the reviewers. Any product that may be evaluated in this article, or claim that may be made by its manufacturer, is not guaranteed or endorsed by the publisher.

## Supplementary Material

Supplementary Table 1 Area of the traces of isoleucine and arginine obtained by MRM.

## References

[B1] Alloul A. Wuyts S. Lebeer S. Vlaeminck S. E. (2019). Volatile fatty acids impacting phototrophic growth kinetics of purple bacteria: paving the way for protein production on fermented wastewater. *Water Res.* 152 138–147. 10.1016/j.watres.2018.12.025 30665160

[B2] Bayon-Vicente G. Wattiez R. Leroy B. (2020a). Global proteomic analysis reveals high light intensity adaptation strategies and polyhydroxyalkanoate production in rhodospirillum rubrum cultivated with acetate as carbon source. *Front. Microbiol.* 11:464. 10.3389/fmicb.2020.00464 32269553 PMC7109303

[B3] Bayon-Vicente G. Zarbo S. Deutschbauer A. Wattiez R. Leroy B. (2020b). Photoheterotrophic assimilation of valerate and associated polyhydroxyalkanoate production by *Rhodospirillum rubrum*. *Appl. Environ. Microbiol.* 86:e00901. 32651203 10.1128/AEM.00901-20 PMC7480388

[B4] Beaufay F. Coppine J. Hallez R. (2021). When the metabolism meets the cell cycle in bacteria. *Curr Opin Microbiol* 60 104–113. 10.1016/j.mib.2021.02.006 33677348

[B5] Berg I. A. Ivanovsky R. N. (2009). Enzymes of the citramalate Cycle in *Rhodospirillum Rubrum*. *Mikrobiologiia* 78 16–24. 10.1134/s0026261709010032 19334594

[B6] Bradford M. M. (1976). A rapid and sensitive method for the quantitation of microgram quantities of protein utilizing the principle of protein-dye binding. *Anal. Biochem.* 72 248–254. 942051 10.1016/0003-2697(76)90527-3

[B7] De Meur Q. Deutschbauer A. Koch M. Bayon-Vicente G. Cabecas Segura P. Wattiez R. (2020). New perspectives on butyrate assimilation in *Rhodospirillum rubrum* s1h under photoheterotrophic conditions. *BMC Microbiol.* 20:126. 10.1186/s12866-020-01814-7 32434546 PMC7238569

[B8] De Meur Q. Deutschbauer A. Koch M. Wattiez R. Leroy B. (2018). Genetic plasticity and ethylmalonyl coenzyme a pathway during acetate assimilation in *Rhodospirillum Rubrum* S1H under photoheterotrophic conditions. *Appl. Environ. Microbiol.* 84:e02038. 10.1128/AEM.02038-17 PMC5772224 29180364

[B9] Elišáková V. Pátek M. Holátko J. Nesvera J. Leyval D. Goergen J.-L. (2005). Feedback-resistant acetohydroxy acid synthase increases valine production in *Corynebacterium glutamicum*. *Appl. Environ. Microbiol.* 71 207–213. 15640189 10.1128/AEM.71.1.207-213.2005 PMC544200

[B10] Erb T. J. Fuchs G. Alber B. E. (2009). (2S)- Methylsuccinyl-CoA dehydrogenase closes the ethylmalonyl-CoA pathway for Acetyl-CoA assimilation. *Mol. Microbiol.* 73 992–1008. 19703103 10.1111/j.1365-2958.2009.06837.x

[B11] Erb T. J. Rétey J. Fuchs G. Alber B. E. (2008). Ethylmalonyl-CoA mutase from rhodobacter sphaeroides defines a new subclade of coenzyme B12-dependent acyl-CoA mutases. *J. Biol. Chem.* 283 32283–32293. 18819910 10.1074/jbc.M805527200

[B12] Erickson D. L. Lines J. L. Pesci E. C. Venturi V. Storey D. G. (2004). *Pseudomonas Aeruginosa* RelA contributes to virulence in drosophila melanogaster. *Infect. Immun.* 72 5638–5645. 15385461 10.1128/IAI.72.10.5638-5645.2004 PMC517598

[B13] Eydallin G. Viale A. M. Morán-Zorzano M. T. Muñoz F. J. Montero M. Baroja-Fernández E. (2007). Genome-wide screening of genes affecting glycogen metabolism in *Escherichia Coli* K-12. *FEBS Lett.* 581 2947–2953. 17543954 10.1016/j.febslet.2007.05.044

[B14] Fang M. Bauer C. E. (2018). Regulation of stringent factor by branched-chain amino acids. *Proc. Natl. Acad. Sci. U.S.A.* 115 6446–6451. 29866825 10.1073/pnas.1803220115 PMC6016800

[B15] Golomysova A. Gomelsky M. Ivanov P. S. (2010). Flux balance analysis of photoheterotrophic growth of purple nonsulfur bacteria relevant to biohydrogen production. *Int. J. Hydrog. Energy* 35 12751–12760. 10.1016/j.ijhydene.2010.08.133

[B16] Gordon G. C. McKinlay J. B. (2014). Calvin cycle mutants of photoheterotrophic purple nonsulfur bacteria fail to grow due to an electron imbalance rather than toxic metabolite accumulation. *J. Bacteriol.* 196 1231–1237. 10.1128/JB.01299-13 24415727 PMC3957710

[B17] He H. Cooper J. N. Mishra A. Raskin D. M. (2012). Stringent response regulation of biofilm formation in *Vibrio Cholerae*. *J. Bacteriol.* 194 2962–2972. 10.1128/JB.00014-12 22467780 PMC3370634

[B18] Ivanovsky R. N. Krasilnikova E. N. Berg I. A. (1997). A proposed citramalate cycle for acetate assimilation in the purple non- sulfur bacterium *Rhodospirillum rubrum*. *FEMS Microbiol. Lett.* 153 399–404. 10.1111/j.1574-6968.1997.tb12602.x

[B19] Klamt S. Grammel H. Straube R. Ghosh R. Gilles E. D. (2008). Modeling the electron transport chain of purple non-sulfur bacteria. *Mol. Syst. Biol.* 4:156. 10.1038/msb4100191 18197174 PMC2238716

[B20] Leroy B. De Meur Q. Moulin C. Wegria G. Wattiez R. (2015). New insight into the photoheterotrophic growth of the isocytrate lyase-lacking purple bacterium *Rhodospirillum Rubrum* on acetate. *Microbiology* 161 1061–1072. 10.1099/mic.0.000067 25737481

[B21] Magnusson L. U. Farewell A. Nyström T. (2005). PpGpp: a global regulator in *Escherichia Coli*. *Trends Microbiol.* 13 236–242. 10.1016/j.tim.2005.03.008 15866041

[B22] Magnusson L. U. Nyström T. Farewell A. (2003). Underproduction of Σ70 mimics a stringent response: a proteome approach. *J. Biol. Chem.* 278 968–973. 10.1074/jbc.M209881200 12421813

[B23] Masuda S. Bauer C. E. (2004). Null mutation of HvrA compensates for loss of an essential rela/spot-like gene in *Rhodobacter Capsulatus*. *J. Bacteriol.* 186 235–239. 10.1128/JB.186.1.235-239.2004 14679243 PMC303453

[B24] McCully A. L. Onyeziri M. C. LaSarre B. Gliessman J. R. McKinlay J. B. (2020). Reductive tricarboxylic acid cycle enzymes and reductive amino acid synthesis pathways contribute to electron balance in a rhodospirillum rubrum calvin-cycle mutant. *Microbiology* 166 199–211. 10.1099/mic.0.000877 31774392

[B25] McKinlay J. B. Harwood C. S. (2010). Carbon dioxide fixation as a central redox cofactor recycling mechanism in bacteria. *Proc. Natl. Acad. Sci. U.S.A.* 107 11669–11675. 10.1073/pnas.1006175107 20558750 PMC2900684

[B26] McKinlay J. B. Harwood C. S. (2011). Calvin cycle flux, pathway constraints, and substrate oxidation state together determine the h2 biofuel yield in photoheterotrophic bacteria. *Mbio* 2:e00323. 10.1128/mBio.00323-10 21427286 PMC3063381

[B27] Mittenhuber G. (2001). Comparative genomics and evolution of genes encoding bacterial (p)PpGpp Synthetases/Hydrolases (the Rel, RelA and SpoT Proteins). *J. Mol. Microbiol. Biotechnol.* 3 585–600. 11545276

[B28] Muhitch M. J. (1988). Acetolactate synthase activity in developing maize (Zea Mays L.) Kernels. *Plant Physiol.* 86 23–27. 10.1104/pp.86.1.23 16665871 PMC1054421

[B29] Osumi T. Katsuki H. (1977). A novel pathway for L-citramalate synthesis in *Rhodospirillum Rubrum*. *J. Biochem.* 81 771–778. 10.1093/oxfordjournals.jbchem.a131515 405381

[B30] Paul B. J. Berkmen M. B. Gourse R. L. (2005). DksA potentiates direct activation of amino acid promoters by PpGpp. *Proc. Natl. Acad. Sci. U.S.A.* 102 7823–7828. 10.1073/pnas.0501170102 15899978 PMC1142371

[B31] Pizarro-Cerdá J. Tedin K. (2004). The bacterial signal molecule, PpGpp, regulates *Salmonella* virulence gene expression. *Mol. Microbiol.* 52 1827–1844. 10.1111/j.1365-2958.2004.04122.x 15186428

[B32] Rizk M. L. Laguna R. Smith K. M. Tabita F. R. Liao J. C. (2011). Redox homeostasis phenotypes in rubisco-deficient rhodobacter sphaeroides via ensemble modeling. *Biotechnol. Prog.* 27 15–22. 10.1002/btpr.506 20939096

[B33] Ronneau S. Petit K. De Bolle X. Hallez R. (2016). Phosphotransferase-dependent accumulation of (p)PpGpp in response to glutamine deprivation in *Caulobacter crescentus*. *Nat. Commun.* 7:11423. 10.1038/ncomms11423 27109061 PMC4848567

[B34] Ruiz J. A. López N. I. Méndez B. S. (2004). RpoS gene expression in carbon-starved cultures of the polyhydroxyalkanoate-accumulating species *Pseudomonas* Oleovorans. *Curr. Microbiol.* 48 396–400. 10.1007/s00284-003-4183-5 15170232

[B35] Shimizu M. Fujii T. Masuo S. Takaya N. (2010). Mechanism of de novo branched-chain amino acid synthesis as an alternative electron sink in hypoxic aspergillus nidulans cells. *Appl. Environ. Microbiol.* 76 1507–1515. 10.1128/AEM.02135-09 20081005 PMC2832390

[B36] Tanaka Y. (2003). Properties of acetolactate synthase from sulfonylurea-resistant scirpus juncoides roxb. var. ohwianus t. koyama. *Pestic. Biochem. Physiol.* 77 147–153. 10.1016/j.pestbp.2003.07.005

[B37] Wang D. Zhang Y. Welch E. Li J. Roberts G. P. (2010). Elimination of rubisco alters the regulation of nitrogenase activity and increases hydrogen production in *Rhodospirillum Rubrum*. *Int. J. Hydrog. Energy* 35 7377–7385. 10.1016/j.ijhydene.2010.04.183 20652089 PMC2905822

[B38] Westerfeldt W. W. (1945). A colorimetric determination of blood acetoin. *J. Biol. Chem.* 161 495–502. 10.1016/S0021-9258(17)41484-0 21006932

[B39] Xiao H. Kalman M. Ikehara K. Zemel S. Glaser G. Cashel M. (1991). Residual guanosine 3’,5’-bispyrophosphate synthetic activity of RelA null mutants can be eliminated by spot null mutations. *J. Biol. Chem.* 266 5980–5990. 10.1016/S0021-9258(19)67694-5 2005134

